# Blunted midbrain reward activation during smoking withdrawal: a preliminary study

**DOI:** 10.3389/fphar.2024.1426506

**Published:** 2024-07-02

**Authors:** A. A. Conti, S. Tolomeo, A. Baldacchino, J. D. Steele

**Affiliations:** ^1^ Department of Child and Adolescent Psychiatry, Institute of Psychiatry, Psychology and Neuroscience, King’s College London, London, United Kingdom; ^2^ Population and Behavioural Science Division, School of Medicine, University of St Andrews, St. Andrews, United Kingdom; ^3^ Institute of High Performance Computing, Agency for Science, Technology and Research, Singapore, Singapore; ^4^ Division of Imaging Science and Technology, Medical School, University of Dundee, Dundee, United Kingdom

**Keywords:** functional magnetic resonance imaging, Positive Valence System, Negative Valence System, smoking, nicotine withdrawal, nicotine, midbrain, reward

## Abstract

**Introduction:**

Tobacco smoking is the leading preventable cause of death, causing more than six million deaths annually worldwide, mainly due to cardiovascular disease and cancer. Many habitual smokers try to stop smoking but only about 7% are successful, despite widespread knowledge of the risks. Development of addiction to a range of substances is associated with progressive blunting of brain reward responses and sensitisation of stress responses, as described by the allostasis theory of addiction. There is pre-clinical evidence from rodents for a dramatic decrease in brain reward function during nicotine withdrawal.

**Methods:**

Here we tested the hypothesis that habitual smokers would also exhibit blunted reward function during nicotine withdrawal using a decision-making task and fMRI.

**Results:**

Our findings supported this hypothesis, with midbrain reward-related responses particularly blunted. We also tested the hypothesis that smokers with a longer duration of smoking would have more pronounced abnormalities. Contrary to expectations, we found that a shorter duration of smoking in younger smokers was associated with the most marked abnormalities, with blunted midbrain reward related activation including the dopaminergic ventral tegmental area.

**Discussion:**

Given the substantial mortality associated with smoking, and the small percent of people who manage to achieve sustained abstinence, further translational studies on nicotine addiction mechanisms are indicated.

## Introduction

Tobacco smoking is the leading preventable cause of death, with more than six million deaths annually worldwide; on average, smokers lose 10 years of life compared to people who have never smoked (Kondo et al., 2019). Smoking-related deaths are predominately caused by cancer and cardiovascular disease, with the latter causing one-third of all smoking-related deaths (Kondo et al., 2019). Lung cancer is the leading cause of cancer-related deaths, and local lung cancer mortality largely follows the local geographical tobacco smoking prevalence (Barta et al., 2019).

Knowledge of smoking-related cancer and cardiovascular risks is widespread in society worldwide. Many habitual tobacco smokers attempt to permanently stop, but only approximately 7% are successful (Pierce, 2022), and the majority of relapses occur within the first few days of attempting to stop smoking. Therefore, knowledge of substantial risks is not enough to make most people stop smoking. Whilst some people mistakenly regard continued smoking as a free choice, the National Health Service (NHS) reports that the main reason people continue to smoke tobacco is because of the addictive nature of nicotine present in tobacco products, such as cigarettes (https://www.nhs.uk/conditions/stop-smoking-treatments/).

Considerable pre-clinical evidence from studies on animals shows that development of addiction to a range of substances, including alcohol and opioids, is associated with progressive blunting of brain reward responses and increasing sensitisation of stress responses (Koob, 2013). Whilst nicotine is less effective as a positive reinforcer than other drugs of abuse in non-dependent animals, nicotine withdrawal symptoms in dependent humans include low mood, anxiety, anger/irritability, and craving (Epping-Jordan et al., 1998; Conti et al., 2020), contributing to the addictive properties of nicotine (Markou et al., 1998). Notably, nicotine withdrawal in rodents has been reported to be associated with a “dramatic decrease” in brain reward function, which lasted for 4 days (Epping-Jordan et al., 1998). The study conducted on rodents used an invasive method to measure relative reward thresholds involving implanted electrodes (Epping-Jordan et al., 1998). In this study, our aim was to test for changes in the reward threshold in humans using a functional magnetic resonance imaging (fMRI)-based non-invasive measurement of reward function.

Ellison reported that in rodents, major addictive stimulants such as cocaine and amphetamine cause degeneration in the fasciculus retroflexus, which carries much of the descending inhibitory control from the forebrain via the lateral habenula to midbrain dopaminergic and serotonergic neurons, linked to drug-related behavioural changes (Ellison, 2002). Nicotine, at plasma concentrations relevant to human smokers, was also found to cause an “extraordinary selective” degeneration of cholinergic fasciculus retroflexus tracts, specifically from the medial habenula, which projects to the midbrain dopaminergic ventral tegmental area (VTA), implying a link between addiction caused by major stimulant drugs and addiction caused by nicotine (Ellison, 2002). Decreased reward function during nicotine withdrawal in rodents is comparable to decreased reward function caused by major drugs that cause dependency, which may be an important factor contributing to relapse to tobacco use in humans (Epping-Jordan et al., 1998).

Various factors affect the impact of habitual smoking. Nicotine is more problematic for younger smokers as the brain is still undergoing significant developmental change. Yuan et al. (2015) identified persistent acetylcholine receptor upregulation and greater acetylcholine disruption in adolescent rodents than in adult rodents. The duration of habitual tobacco smoking is another relevant factor, which is important with regard to carcinogenesis and adverse effects on the cardiovascular system. However, the effect of a longer versus shorter duration of habitual smoking on the reward system is largely unknown, and studies investigating the age at the onset of smoking or nicotine exposure may be confounded by the duration of exposure effects. Pre-clinical studies on rodents tend to be of very short duration compared to the decades of habitual smoking that occurs in humans.

Allostasis theories have been developed from invasive pre-clinical models of human addictions, but similar evidence from invasive studies on humans is not available for ethical reasons, although lower striatal dopamine D2 receptor availability in smokers has been reported using molecular imaging (Wiers et al., 2017). We described a non-invasive fMRI-based approach aligned with the Research Domain Criteria (RDoC) (Insel et al., 2010) focusing on the Positive Valence System (PVS) and Negative Valence System (NVS), using it to test the allostasis theory-derived hypothesis for human binge alcohol drinkers (Tolomeo et al., 2020) and long-term abstinent, former opioid-dependent, patients (Tolomeo et al., 2022).

Here, we used our fMRI-based approach to test our first hypothesis that nicotine-dependent humans experiencing nicotine withdrawal exhibit blunted PVS reward function and elevated NVS function, consistent with allostasis theory predictions. Our second hypothesis was that a longer duration of habitual smoking would be associated with more pronounced negative effects on the reward system. It should be noted that we did not test hypotheses about smoking-related *cues* (McClernon et al., 2005; Englemann et al., 2012). Instead, we tested for hypothesised abnormalities of brain reward and aversion responses to nonsmoking-related *outcomes*, so our study design and analyses reflected this. We also performed exploratory analyses to investigate whether a longer duration of habitual smoking would result in more severe negative mood symptoms (e.g., anger, depression, anxiety, and anhedonia) and tobacco craving for smokers experiencing nicotine withdrawal.

## Materials and methods

### Participants

Ethical approval for the study was granted by the London Bromley Research Ethics Committee (REC) (REC Reference Number: 19/LO/1176) and the University of St. Andrews Teaching and Research Ethics Committee (UTREC) (UTREC Approval Code: MD14516). Twenty-seven tobacco smokers and 24 matched nonsmoker controls were recruited across the south-east region of Scotland between October 2019 and March 2020 (Conti and Baldacchino, 2021). A group of younger smokers (mean age, 21 years; mean years of habitual smoking, 6 years) and younger nonsmoking controls and a group of older smokers (mean age, 36 years; mean years of habitual smoking, 18 years) and older nonsmoking controls were recruited and matched for the age at the onset of habitual tobacco smoking and use of alcohol. The age at the onset of regular smoking was defined as the age at which participants started smoking ≥5 tobacco cigarettes per day. Smoker participants had to smoke ≥10 cigarettes per day for 2 or more years to be included in the study. Controls had to be lifetime nonsmokers. For inclusion in the study, smokers needed to present a carbon monoxide (CO) level ≥10 ppm and a salivary cotinine level >20 ng/mL, while nonsmokers needed to present a CO level ≤4 ppm and a salivary cotinine level of <20 ng/mL. The presence of illicit substances in participants was tested using urine drug analysis. Participants who were positive for illicit substances were excluded from the study, with the exception of occasional cannabis users (≤2 joints per week). Participants with a significant current and/or previous history of psychiatric and/or neurological illnesses were excluded. Sociodemographic and smoking characteristics of participants are given in Tables 1, 2.

**TABLE 1:** Participant details.

Session 1					
	Younger smokers	Older smokers	Younger nonsmokers	Older nonsmokers	Significance
Sociodemographic characteristics					
N	15	13	14	10	
Age in years (SD)	21.13 (2.23)	36.23 (4.22)	21.57 (1.86)	38.40 (6.56)	Older smokers > younger smokers = *p* < 0.001
					Older smokers > younger nonsmokers = *p* < 0.001
					Older nonsmokers > younger smokers = *p* < 0.001
					Older nonsmokers > younger nonsmokers = *p* < 0.001
Sex (%)	60.0% females	7.70% females	64.29% females	20.0% females	Younger female smokers > older female smokers = *p* < 0.01
	40.0% males	92.30% males	35.71% males	80% males	Younger female nonsmokers > older female nonsmokers = *p* < 0.05
Tobacco smoking characteristics					
Cigarettes smoked x day	13.50 (3.58)	16.80 (3.92)	N/A	N/A	Older smokers > younger smokers = *p* < 0.05
FTND	4.46 (1.50)	5.69 (1.25)	N/A	N/A	Older smokers > younger smokers = *p* < 0.05
Years of smoking	6.10 (3.55)	18.84 (7.10)	N/A	N/A	Older smokers > younger smokers = *p*< 0.001
Pack years	5.40 (3.77)	16.23 (8.02)	N/A	N/A	Older smokers > younger smokers = *p* < 0.05
Age at the onset of regular smoking (years)	15.16 (2.50)	17.38 (3.99)	N/A	N/A	*p* > 0.05
CO level	18.73 (6.09)	25.53 (10.98)	1.14 (0.53)	1.40 (0.51)	Older smokers > younger nonsmokers = *p* < 0.001
					Older smokers > younger nonsmokers = *p* < 0.001
					Older smokers > younger nonsmokers = *p* < 0.001
					Older smokers > younger nonsmokers = *p* < 0.001
Other substance use characteristics					
Units of alcohol consumed x day	0.93 (1.16)	0.38 (0.86)	0.14 (0.36)	0.45 (0.79)	*p* > 0.05
*n* Cannabis smokers	2	2	N/A	N/A	*p* > 0.05

**Note:** Data are presented as the mean and standard deviation (SD) or in percentages (%). Sig1 = significance at *p* < 0.05 in the two-tailed *t*-test. %, percentage; n, number of participants; CO, carbon monoxide; SD, standard deviation; FTND, Fagerström Test for Nicotine Dependence (0–2 = very low dependence, 2–4 = low dependence, 5 = medium dependence, and 6 or more = high dependence).

**TABLE 2:** Participant details.

Session 2 (fMRI)					
	Younger smokers	Older smokers	Younger nonsmokers	Older nonsmokers	Significance
Sociodemographic characteristics					
n	12	11	10	9	
Age in years (SD)	21.50 (2.27)	36.27 (4.60)	21.90 (2.13)	38.55 (6.94)	Older smokers > younger smokers = *p* < 0.001
					Older smokers > younger nonsmokers = *p* < 0.001
					Older nonsmokers > younger smokers = *p* < 0.001
					Older nonsmokers > younger nonsmokers = *p* < 0.001
Sex (%)	66.66% females	9.10% females	60.0% females	22.22% females	Younger female smokers > older female smokers = *p* < 0.01
	33.33% males	90.90% males	40.0% males	77.78% males	
Tobacco smoking characteristics					
Cigarettes smoked x day	13.79 (3.85)	17.36 (3.99)	N/A	N/A	Older smokers > younger smokers = *p* < 0.05
FTND	4.50 (1.56)	5.81 (1.25)	N/A	N/A	Older smokers > younger smokers = *p* < 0.05
Years of smoking	6.83 (3.56)	18.63 (7.74)	N/A	N/A	Older smokers > younger smokers = *p*< 0.001
Pack years	6.25 (3.69)	16.72 (8.68)	N/A	N/A	Older smokers > younger smokers = *p* < 0.001
Age at the onset of regular smoking (years)	15.20 (2.79)	17.45 (4.34)	N/A	N/A	*p* > 0.05
CO level	3.00 (1.70)	6.63 (2.54)	N/A	N/A	Older smokers > younger smokers = *p* < 0.001
Other substance use characteristics					
Units of alcohol consumed x day	0.75 (0.86)	0.36 (0.92)	0.20 (0.42)	0.22 (0.36)	*p* > 0.05
*n* cannabis smokers	2	2	N/A	N/A	*p* > 0.05

**Note:** Data are presented as the mean and standard deviation (SD) or in percentages (%). Sig1 = significance at *p* < 0.05 in the two-tailed *t*-test. %, percentage; n, number of participants; CO, carbon monoxide; SD, standard deviation; FTND, Fagerström Test for Nicotine Dependence (0–2 = very low dependence, 2–4 = low dependence, 5 = medium dependence, and 6 or more = high dependence).

### Procedures

Participants needed to attend two experimental sessions on 2 separate days. The first session was conducted at the University of St. Andrews School of Medicine and encompassed both screening and experimental procedures. Specifically, both smoker and nonsmoker participants undertook a CO breath test, a cotinine saliva test, and urine drug analysis in addition to other screening assessments described previously (Conti and Baldacchino, 2021; Conti and Baldacchino, 2022). Regarding experimental procedures, smokers completed the abbreviated Profile of Mood States (POMS), the Snaith–Hamilton Pleasure Scale (SHAPS), and the Brief Questionnaire of Smoking Urges (QSU). Furthermore, both smoker and nonsmoker participants performed neurocognitive tests that have been reported previously (Conti and Baldacchino, 2021), but those are outside the scope of this paper. Smokers were instructed to smoke as they wished (*ad libitum* smoking) prior to attending the first experimental session.

The second experimental session was conducted at Ninewells Hospital, Dundee. Smoker participants attended the session in a nicotine-withdrawal state. Specifically, smoker participants were instructed to stop smoking the night before the scanning session at 22:00. Nicotine withdrawal was verified through a CO breath test by utilising a cut-off value of ≤9 ppm in accordance with the Society for Research on Nicotine and Tobacco (SNRT) subcommittee on biochemical verification (Benowitz et al., 2020). The mean hours of smoking withdrawal at the time of scanning were 14.5 h (SD = 1.78). This session involved an fMRI procedure for both smoker and nonsmoker participants. Smoker participants were also asked to complete the POMS, SHAPS, and QSU prior to scanning.

### Measures

#### Abbreviated POMS

The abbreviated POMS is a self-report psychological rating scale designed to measure transient mood states across different mood constructs including tension–anxiety, anger–hostility, and depression–dejection (McNair and Loor, 1971). The POMS has been widely utilised to investigate changes in mood states resulting from nicotine withdrawal (Heffner et al., 2011; Conti et al., 2020). The abbreviated version of the POMS constitutes of 40 items. Participants needed to rate each item on a 5-point Likert scale ranging from 0 (not at all) to 4 (extremely). Scores were computed for each mood construct in addition to a total POMS score. The psychometric properties of the abbreviated POMS scale were investigated by Grove and Prapavessis (1992), showing acceptable validity and high reliability with a mean coefficient of 0.80.

#### Snaith–Hamilton Pleasure Scale

The SHAPS is a 14-item scale designed by Snaith et al. (1995) to measure the ability to experience pleasure from naturally rewarding stimuli (hedonic capacity) during a determined period of time. As stated by Borsini et al. (2020), anhedonia is characterised by deficits in three reward-processing subtypes: reward liking, reward wanting, and reward learning.

The scale does not utilise the Likert scoring method; it has two options: the “disagree” option scores 1 point, while the “agree” option scores 0 point. Thus, participants needed to rate each item on a 4-point scale: “strongly disagree” (1), “disagree” (1), “agree” (0), and “strongly agree” (0). The total score ranges from 0 to 14 (Snaith et al., 1995). A score >2 indicates an “abnormal” hedonic capacity (Snaith et al., 1995). Nakonezny et al. (2010) explored the psychometric properties of the scale, revealing high validity and excellent internal consistency with a coefficient of 0.91.

#### Brief Questionnaire of Smoking Urges

The Brief Questionnaire of Smoking Urges (QSU-Brief) (Cox et al., 2001) is the 10-item version of the QSU instrument developed by Tiffany and Drobes (1991) to measure the magnitude of smoking urges/craving emerging during nicotine withdrawal. As underlined by Cox et al. (2001), the QSU-Brief assesses the multidimensional nature of craving by measuring two distinct factors: “Factor 1 represents a strong desire and intention to smoke, with smoking perceived as rewarding for active smokers, while Factor 2 reflects an anticipation of relief from negative affect and an urgent desire to smoke” (Cox et al., 2001, p.13). Participants needed to rate each item through a 7-point Likert scale ranging from 1 (strongly disagree) to 7 (strongly agree). Several studies investigated the psychometric properties of the QSU-Brief, revealing good internal reliability and validity (Toll et al., 2006; Littel et al., 2011).

### Neuroimaging

For each participant, functional whole-brain images were acquired using a 3T Siemens Tim Trio scanner at the Clinical Research Centre, Ninewells Hospital, Dundee. A total of 37 slices were obtained per volume, with an echo-planar imaging sequence comprising a repetition time (TR) of 2.5 s, echo time (TE) of 30 ms, flip angle of 90°, field of view of 22.4 cm, 64 × 64 matrix, and a voxel size of 3.5 × 3.5 × 3.5 mm. Images were pre-processed using statistical parametric mapping (SPM12) (Friston, 1994), which comprised realignment to the initial scan in each participant’s time series. The mean realigned image was calculated and then used to determine the spatial normalisation transformations, which were applied to the realigned images smoothed using an 8-mm FWHM Gaussian kernel.

### fMRI paradigm

**FIGURE 1:**
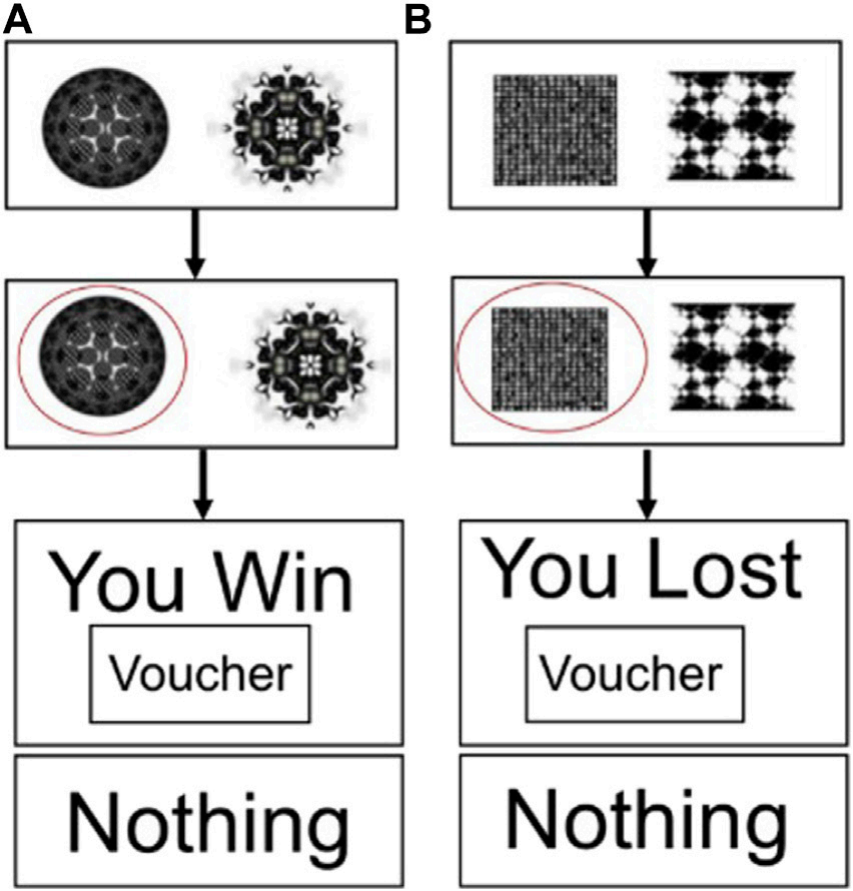
Decision-making task used during fMRI. **(A)** Reward–gain trials and **(B)** loss–avoidance trials.

Figure 1 shows the reward–gain and loss avoidance instrumental learning task used during fMRI (Tolomeo et al., 2020; Tolomeo et al., 2022). Before scanning, all participants had a brief training session on the task on a PC, which used different stimuli from those used in the scanner. The task had three possible outcomes: rewarding (“win”), aversive (“lose”), and neither win or lose (“nothing”). Participants were informed that the aim of the task was to maximise winning and avoid losing points (“vouchers’) as much as possible: win–gain trials had the possible outcomes “win” or “nothing,” and loss–avoidance trials had the possible outcomes “lose” or “nothing.” One pair of fractal images were associated with each type of outcome (win or lose), and the association between a given pair of fractal images and outcomes was randomised across participants. The probability of win/loss fractal pairs had a fixed high probability (70%) and a fixed low probability (30%). Each session had 90 trials, with each session lasting 13 min in total and four sessions per subject. The reward–gain and loss avoidance trials were presented in a pseudo-random order.

### Statistical analysis

Factorial ANOVAs were utilised to compare younger smokers, older smokers, younger nonsmokers, and older nonsmokers in relation to age, daily alcohol usage, and tobacco smoking variables (n° cigarettes smoked per day, years of smoking, Fagerström Test for Nicotine Dependence [FTND] scores, and age at the onset of regular smoking) for both experimental sessions. Chi-squared (χ^2^) tests of associations were instead utilised to investigate differences in relation to biological sex and n° of occasional cannabis users between the four groups of participants. Independent-sample *t*-tests and chi-squared (χ^2^) tests of associations were utilised to compare all smokers (younger + older groups) against all nonsmokers (younger + older groups) in relation to age, biological sex, and units of alcohol consumed per day as per our previous paper (Conti and Baldacchino, 2021).

Mixed ANOVAs were utilised to investigate whether a longer duration of habitual smoking resulted in more severe negative moods and tobacco-craving symptoms for smokers experiencing nicotine withdrawal. Particularly, the smoker group (younger vs. older) was inserted as a between-subject factor, while the smoking condition (*ad libitum* smoking vs. nicotine withdrawal) was inserted as a within-subject factor. Main effect comparisons were conducted with Bonferroni adjustment to control for type-1 error. The significance level was set at *p* < 0.05. SPSS v. 28 (SPSS Inc., United States) was utilised for this part of the analysis.

**FIGURE 2:**
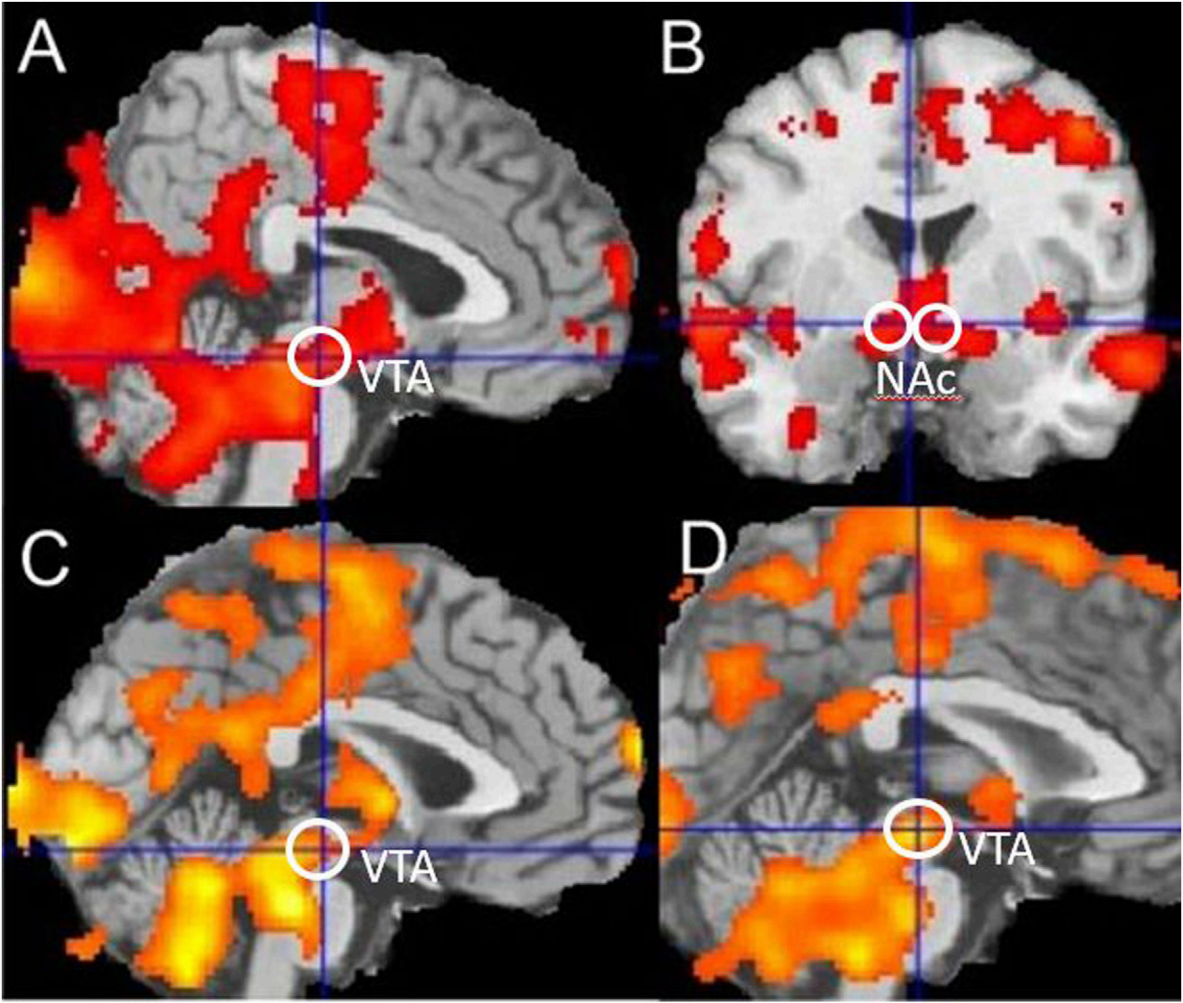
Reward-related activation in smokers and controls. **(A,B)** Reward-linked activation for all nonsmoker controls, **(C)** blunted reward-related activation in all smokers during withdrawal compared to nonsmoker controls, and **(D)** blunted reward-related activation in younger smokers during withdrawal compared to younger nonsmoker controls. Ventral Tegmental Area (VTA) and Nucleus Accumbens (NAc) regions are indicated. Significance as *p* < 0.05 corrected is indicated by the presence of any colour in the figure.

For fMRI event-related, random-effects analyses, data on each subject were analysed separately (first-level analyses) before summary “beta” images were tested at a group level (second-level analyses). First-level within-subject analyses focused on the feedback event in the reward–gain (“win” or “nothing”) and loss–avoidance (“loss” or “nothing”) trials. For second-level between-subject analyses, summary “beta” images from the first-level analyses used one-group and two-group *t*-tests. For voxel-based analyses, significance was defined as *p* < 0.05 at a whole-brain, family-wise error-corrected level, comprising a simultaneous requirement for a voxel threshold (*p* < 0.05) and a minimum cluster extent (120 voxels) identified using the popular Monte Carlo method (Slotnick et al., 2003). In Figure 2, significance as *p* < 0.05 cluster corrected is indicated by the presence of any colour. Further interpretation is not possible because a cluster-based significance correction method does not allow inferences about the relative significance of any coloured region, although SPM shows lighter colours where voxel t-values are numerically larger.

To test for any specific change in VTA reward-related activity, we used an *a priori* defined volume of interest defined by two previous studies (Gu et al., 2010; Hadley et al., 2014), comprising a 6-mm-diameter sphere centred at (0, −16, −7). A binary mask was created using MarsBaR (Brett et al., 2006; Brett et al., 2002) for this volume, and SPM12 was used to calculate the mean whitened and filtered beta values for each subject. The null hypothesis of no difference was then tested using JASP (https://jasp-stats.org/).

## Results

### Subjects

The sociodemographic and smoking characteristics of participants are given in Tables 1, 2. Ten participants (five smokers and five nonsmokers) dropped out from the study before attending session 2. Two smoker participants were excluded from the study prior to session 2 due to failure to maintain nicotine withdrawal as objectively verified by the CO breath test; two participants (one smoker and one nonsmoker) dropped out due to the burden of attending an fMRI session, while the remaining six participants (four nonsmokers and two smokers) did not attend session 2 due to COVID-19 restrictions.

During both sessions (session 1 and session 2), comparison of the younger group of smokers with younger nonsmokers and older smokers with older nonsmokers revealed no significant differences in age (*p* > 0.05). Similarly, no significant biological sex differences were identified between young smokers and young nonsmokers and between older smokers and older nonsmokers (*p* > 0.05). However, the % of females in the young smoker and young nonsmoker groups was significantly higher than that in the older smoker and older nonsmoker groups (*p* < 0.05).

Younger and older smokers had no statistically significant differences regarding the age at the onset of regular smoking (*p* > 0.05). Furthermore, there were no statistically significant differences between younger and older smokers regarding the units of alcohol consumed per day and the number of occasional cannabis users (*p* > 0.05). No statistically significant differences in alcohol consumption were detected when comparing older smokers with older nonsmokers (*p* > 0.05). The average ages of the younger and older groups of smokers and nonsmokers were significantly different (*p* < 0.001), and the older group of smokers reported longer years of smoking than the younger group (*p* < 0.001). Furthermore, significant differences were identified between the younger group of smokers and the older group of smokers in relation to number of cigarettes smoked daily, pack years, and severity of nicotine dependence (as assessed by the FTND) (*p* < 0.05). Specifically, the older group of smokers reported a greater number of cigarettes smoked daily, higher pack years, and more severe nicotine dependence than the younger group of smokers.

No significant differences were detected when comparing all smokers with all nonsmokers in relation to age, biological sex, and units of alcohol consumed per day during both experimental sessions as reported in our previous study (Conti and Baldacchino, 2021).

### fMRI paradigm

#### Behavioural analyses

There were no significant differences for rewards gained and losses avoided between groups. This meant that the groups were well-balanced with regard to their behaviour during fMRI, which is important for interpreting fMRI results.

#### Positive valence system

For the nonsmoker control groups combined, using a one-group *t*-test, there was significant reward-related activation in the midbrain (−4, −20, −12), *t* = 4.14, and bilateral nucleus accumbens (NAC) (−4, 0, −6), *t* = 3.59 (14, 10, −4) and *t* = 3.12, as shown in Figure 2. This is consistent with many previous independent studies (Johnston et al., 2015; Gradin et al., 2011). Comparing the younger and older nonsmoker control groups combined, with the younger and older smoking groups combined, using a two-group *t*-test showed that there was blunted reward-related activation in the midbrain (−6, −20, −12), *t* = 3.03, and significant blunted reward-related activation in the bilateral nucleus accumbens (−10, 4, −8), *t* = 2.50 (14, 0, −12) and *t* = 2.7 of smokers. Comparing only younger nonsmoker controls matched to younger smokers, there was significant blunted reward-related activation in the midbrain (−2, −22, −10), *t* = 3.43, and significant blunted reward-related activation in the nucleus accumbens (−10, 6, −6), *t* = 3.14, of younger smokers. Comparing younger nonsmokers to older nonsmokers showed that there was reward-related activation in the nucleus accumbens (−10, 8, 0), *t* = 2.76, and no difference in VTA activation. Comparing only older smokers to older nonsmoker controls showed that there was no significant difference in reward-related activations. Using the VTA volume of interest, significantly blunted reward-related activation was identified (*t* = 2.2, df = 18, *p* = 0.04) for younger smokers compared to matched younger nonsmoker controls.

#### Negative valence system

Regarding loss-related activations, there were no significant differences when comparing groups.

#### Exploratory analyses—mood and craving measures

Regarding anhedonia (SHAPS scores), there was no statistically significant interaction between the smoker groups (younger smokers vs. older smokers) and smoking conditions (*ad libitum* smoking vs. nicotine withdrawal) [F(1, 21) = 0.20, *p* = 0.65, partial η2 = 0.01]. However, the main effect of smoking conditions showed a statistically significant difference in the mean SHAPS scores between *ad libitum* smoking and nicotine withdrawal [F(1, 21) = 10.18, *p* < .005, partial η2 = 0.32]. Similarly, no statistically significant interaction was identified between the smoker groups and smoking conditions regarding anxiety [F(1, 21) = 0.16, *p* = 0.69, partial η2 = 0.00], anger [F(1, 21) = 0.30, *p* = 0.59, partial η2 = 0.01], depression [F(1, 21) = 3.03, *p* = 0.09, partial η2 = 0.12], and the total POMS score [F(1, 21) = 0.01, *p* = 0.89, partial η2 = 0.00]. Nonetheless, the main effect of smoking conditions showed a statistically significant difference in mean anxiety [F(1, 21) = 20.66, *p* < .001, partial η2 = 0.49], anger [F(1, 21) = 22.56, *p* < .001, partial η2 = 0.51], depression [F(1, 21) = 4.72, *p* < 0.05, partial η2 = 0.18], and total POMS scores [F(1, 21) = 17.26, *p* < 0.001, partial η2 = 0.45] between *ad libitum* smoking and nicotine withdrawal. These results suggest that the combined groups of smokers experienced an increase in negative mood states including anxiety, anger, depression, and anhedonia when they were nicotine-deprived for 14.5 h (mean) during experimental session 2.

No statistically significant interaction was identified between the smoker groups and smoking conditions regarding QSU factor 1 [F(1, 21) = 2.97, *p* = 0.09, partial η2 = 0.12] and QSU factor 2 [F(1, 21) = 0.47, *p* = 0.49, partial η2 = 0.02]. As per the previously reported mood measures, the main effect of smoking conditions showed a statistically significant difference in mean QSU factor 1 scores [F(1, 21) = 36.80, *p* < 0.001, partial η2 = 0.63] and QSU factor 2 scores [F(1, 21) = 44.47, *p* < 0.001, partial η2 = 0.67] between *ad libitum* smoking and nicotine withdrawal. Therefore, these results suggest that the combined groups of smokers experienced a surge in tobacco cravings and a strong desire to smoke to a) experience the pleasurable/rewarding effects of smoking (QSU factor 1) and b) relieve negative mood symptoms (QSU factor 2) while they were nicotine-deprived for 14.5 h (Mean) during experimental session 2.

## Discussion

Based on the pre-clinical allostasis theory proposed by Koob (2013) and the investigation of nicotine-dependent rodents during nicotine withdrawal (Epping-Jordan et al., 1998), we tested the hypothesis that nicotine-dependent humans would exhibit blunted PVS function (blunted reward-linked signals) and increased NVS function (increased loss-linked signals) during nicotine withdrawal. Our results supported the hypothesis that nicotine-dependent humans exhibit blunted PVS function during nicotine withdrawal (14.5 mean h since the last smoked cigarette). However, we did not find evidence for elevated NVS responses during nicotine withdrawal. We also tested whether a longer duration of smoking was associated with more pronounced abnormalities. Contrary to predictions, we found more pronounced reward signal blunting in younger smokers with a shorter smoking history. Exploratory analyses showed that the combined groups of smokers experienced an increase in negative mood states (anhedonia, depression, anxiety, and anger) and craving symptoms during nicotine withdrawal. However, older habitual smokers with a longer smoking history (and a higher level of nicotine dependence) did not experience more severe withdrawal symptoms compared to younger smokers.

The primary objective of the present study was to test whether there was evidence for blunting of the reward system of human habitual tobacco smokers during withdrawal, as predicted by a pre-clinical rodent study on nicotine withdrawal (Epping-Jordan et al., 1998). In that study, rodent reward thresholds were measured using intracranial self-stimulation (ICSS), an instrumental reward learning paradigm, whereby rodents learn to continuously respond (e.g., by lever pressing) to electrical stimulation of electrodes implanted in a region such as the posterior–lateral hypothalamus (Epping-Jordan et al., 1998), which is a major part of the reward system in all animals. ICSS is an established method for testing reward system function in all animals including humans (Rolls, 1975). It should be noted that ICSS brain stimulation is electrical and not by infusion of nicotine; the procedure did not involve cues for lever pressing to deliver ICSS outcomes, and functional connectivity between different rodent brain regions was not measured (Epping-Jordan et al., 1998).

For the present study, we used an instrumental reward learning and loss avoidance learning task that we have used for studies on depressive illnesses (Johnston et al., 2015), opioid dependency (Gradin et al., 2014; Tolomeo et al., 2022), and binge alcohol drinking (Tolomeo et al., 2020; 2023). Subjects had to learn to choose between two pairs of nonsmoking-related visual stimuli to maximise rewards and avoid losses. The events of interest were the times when the subjects learnt the outcomes of their decisions, e.g., “you win” or “nothing.” As described in a series of previous studies, this contrast provides a non-invasive measure of brain reward function (Steele et al., 2007; Gradin et al., 2014; Johnston et al., 2015; Tolomeo et al., 2022). It is important to note that the reward signal at the outcome time is different from the signal at the time the pairs of stimuli are presented, and a choice has to be made, the latter being the expected reward value signal (Tolomeo et al., 2023). Similarly, studies measuring brain responses to already learned drug cues (e.g., McClernon et al., 2005) test hypotheses different from those tested here (Tolomeo et al., 2023). Additionally, fMRI brain connectivity studies test different hypotheses and measure different brain signals.

Preclinical studies (Koob and Schulkin, 2019) and human addiction studies using positron emission tomography (Wiers et al., 2017; Goldstein and Volkow, 2002) have provided considerable evidence that addiction to a variety of substances in animals and humans involves a shift from positive reinforcement to negative reinforcement. The RDoC were designed to link subjective symptoms to specific brain functions and can also facilitate forward and reverse translation between preclinical studies on animals and non-invasive studies on humans (Tolomeo et al., 2020). We have used this approach to test for blunted PVS brain responses and elevated NVS brain responses in alcohol binge drinkers (Tolomeo et al., 2020) and long-term abstinent, former opioid-dependent patients (Tolomeo et al., 2022), reporting results consistent with predictions.

Abstinence syndromes in dependent rodents have been observed after stopping alcohol, opiates, other sedatives, and nicotine (Epping-Jordan et al., 1998). In the case of nicotine, this occurs spontaneously and can also be precipitated by nicotinic receptor antagonists, reversed by nicotine administration (Epping-Jordan et al., 1998). Subcutaneous nicotine administration to rodents was used to maintain stable plasma nicotine concentrations, comparable to those reported for human tobacco smokers consuming 30 cigarettes per day, and thresholds of electrical ICSS to the rodent posterior lateral hypothalamus were measured. During rodent nicotine withdrawal, significant elevations of reward thresholds were reported, corresponding to blunted reward function, peaking at 6–8 h after withdrawal, with reward thresholds exceeding 140% baseline values (Epping-Jordan et al., 1998). Consistent with this, we found blunted reward function in humans and a diminished capacity to experience natural rewarding stimuli (i.e., anhedonia as measured by the SHAPS) during nicotine withdrawal (14.5 h).

In the subgroup analysis, contrary to expectations, we found more pronounced reward system blunting in habitual younger tobacco smokers, which implies a shorter duration of exposure to nicotine, and these marked reward system abnormalities could then be due to increased neurotoxic effects of nicotine at a younger age, as has been noted in animal studies (Yuan et al., 2015). However, this interpretation appears less likely as the older and younger groups of smokers were well-matched for the age at the onset of smoking.

Blunting of reward-linked activation in a broad midbrain region included a VTA volume defined by two independent studies, one reporting significant VTA functional connectivity decreased in chronic cocaine users (Gu et al., 2010) and the other reporting reduced VTA connectivity in schizophrenic patients, which was normalised with the response to antipsychotic medication (Hadley et al., 2014). Tobacco smoking is very common in people experiencing schizophrenia, and Ellison argued that the highly specific nicotine-induced degeneration of the fasciculus retroflexus projection to the VTA dopaminergic neurons could interact with the vulnerability to develop schizophrenia and other severe neuropsychiatric illnesses (Ellison, 2002). Consistent with this, we found marked blunting of midbrain reward-related activation in the younger group of smokers in a region that included the VTA.

The results of the exploratory analyses are in line with previous meta-analytic evidence showing an increase in negative mood symptoms during acute nicotine withdrawal (within the first 24 h after smoking cessation) (Conti et al., 2020). Smokers also experienced a surge in cravings to relieve negative mood symptoms (QSU factor 1) and to experience the pleasurable effect of smoking (QSU factor 2). However, no group-by-time interactions were identified regarding these mood and craving symptoms. This may be related to the small sample size as the minimum number of subjects needed to detect group-by-time interactions should be four-fold the number of subjects needed to detect main effects (Heo and Leon, 2010; Guo et al., 2013). Indeed, the small sample size of this study may be considered its main limitation. However, it is important to underline that the present work is a preliminary study, and future research is warranted to replicate these results in a larger number of participants. It should also be noted that the recruitment procedures for this study were hampered by the COVID-19 pandemic, which also posed a challenge for matching the smoker and nonsmoker groups. While the smoker groups were well-matched to the nonsmoker control groups (younger smokers vs. younger nonsmokers, older smokers vs. older nonsmokers, and combined groups of smokers vs. combined groups of nonsmokers), both younger groups of smokers and nonsmokers had more female participants in comparison to older groups of smokers and nonsmokers. It is well known that female smokers experience more severe withdrawal symptoms and have more difficulties in quitting smoking compared to male smokers (e.g., Bjornson et al., 1995; Rojas et al., 1998; Conti et al., 2020). Furthermore, neuroimaging studies have reported sex differences pertaining to the structure and the functional connectivity of the smokers’ frontostriatal system (McCarthy et al., 2019; Lin et al., 2020). Therefore, the possibility that the fMRI findings could have been influenced by the greater percentage of women in the group with younger smokers cannot be excluded.

Another important limitation is the lack of a group with young smokers with a longer smoking history and a group with older smokers with a shorter smoking history. The inclusion and comparisons of these groups, in addition to the comparison between younger smokers with a shorter smoking history and younger smokers with a longer smoking history, would have helped determine whether the blunting of the reward system identified in the current preliminary study is, indeed, independent from the duration of tobacco consumption. For this reason, the findings of this preliminary study should be considered with caution, and the inclusion of these groups, in addition to a pre-withdrawal fMRI session in a nicotine-satiated state, is warranted for future confirmatory studies recruiting a larger sample size.

Despite the above limitations, the numbers of subjects were sufficient to reject the null hypothesis of no blunting in reward function for all smokers and specifically for the younger group of smokers. The limited study size affects the interpretation of null findings. It is possible that a larger study would also find evidence for blunted reward function in older smokers and an increased NVS response during nicotine abstinence. It would not, however, contradict the main findings of this study.

The main strengths of our work are its translation design allowing specific non-invasive tests of allostasis theory, in particular predictions based on findings from invasive ICSS studies on rodents during nicotine withdrawal. To the best of our knowledge, this is the first study in the literature that tested whether the neurobiological effects, as described by Koob’s allostasis theory, in rodents also occur in humans during nicotine withdrawal. Notably, the literature pertaining to neuroimaging on nicotine withdrawal is limited to smoking and reward cue-reactivity studies (for a meta-analysis, see Lin et al., 2021) and functional connectivity studies (Faulkner et al., 2018; Ghahremani et al., 2021). Another strength of this study consists in the stringent inclusion and exclusion criteria and that objective tests (urine analysis, saliva cotinine test, and CO breath test) were utilised to assess the smoking and other substance use status of participants in addition to nicotine withdrawal.

In conclusion, consistent with preclinical studies on rodents, we found evidence for blunted midbrain reward function in humans during nicotine withdrawal, which was more marked in younger smokers with a shorter smoking history. Further human–animal translational studies on different stages of nicotine use and withdrawal are indicated to better understand the additive and neurotoxic effects of nicotine, investigating the different effects of the duration of smoking and age at the onset of smoking, aimed at the development of better treatments for nicotine addiction to reduce its substantial associated mortality and morbidity.

## Data Availability

The raw data supporting the conclusion of this article will be made available by the authors, without undue reservation.
